# What is the effect of noise on the interval timing neural network?

**DOI:** 10.1186/1471-2202-15-S1-P74

**Published:** 2014-07-21

**Authors:** Sorinel A Oprisan, Derek Novo, Catalin V Buhusi

**Affiliations:** 1 Department of Physics and Astronomy, College of Charleston, Charleston, SC 29424, USA; 2 Department of Psychology, Utah State University, Logan, UT 84322, USA

## 

**Figure 1:**
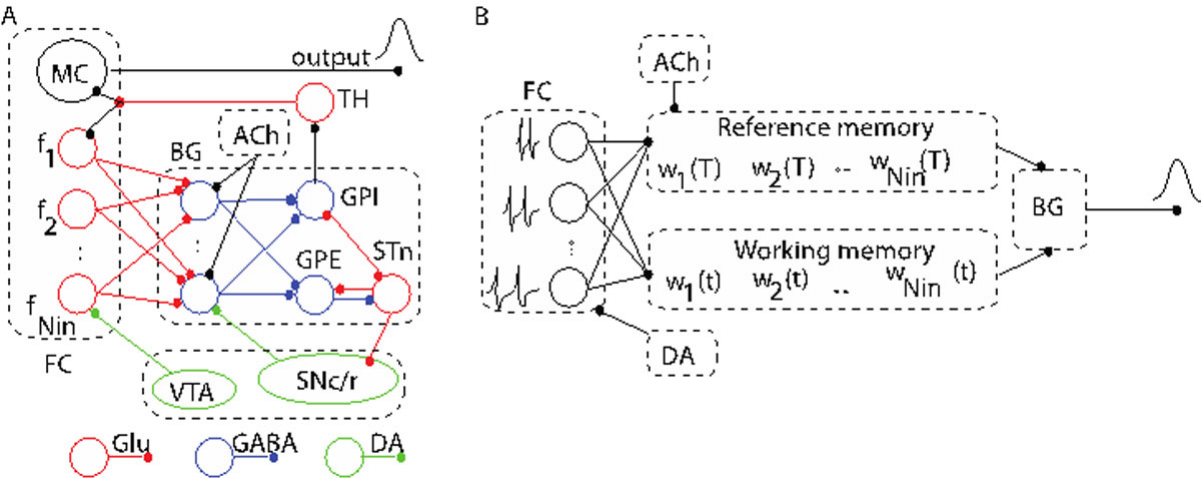
(A) Schematic representation of the two main areas involved in interval timing: frontal cortex (FC) and basal ganglia (BG). (B) SBF model consists of a set of cortical oscillators, a reference memory that stores the state of the system at reinforcement time, a working memory, and a decision block that mimic the activity of BG’s spiny neurons. FC: frontal cortex; MC: motor cortex; BG: basal ganglia; TH: thalamus. GPE: globus pallidus external; GPI: globus pallidus internal; STn: subthalamic nucleus; SNc/r: substantia nigra pars compacta/reticulata; VTA: ventral tegmental area; Glu: glutamate; DA: dopamine; GABA: gamma-aminobutyric acid; ACh: acetylcholine.}

Cognitive processes, such as decision making, rate calculation and planning, are strongly affected by the ability of subjects to perceive durations in the seconds-to-hours range (interval timing) [1]. A classic interval timing paradigm is the peak interval (PI) procedure which consists of interspersed reinforced and probe trials. During the reinforced trials, a stimulus such as a tone or light is turned on to signal the beginning of the to-be-timed interval and the subject’s first response after the criterion time is reinforced. During the probe trials, no reinforcement is given and the stimulus remains on for about three times the criterion time [2]. The mean response rate over a very large number of trials has a Gaussian shape whose peak measures the accuracy of criterion time estimation and the spread of the timing function measures its precision. In the vast majority of species, protocols, and manipulations to date, interval timing is both accurate and time-scale invariant, i.e. time-estimation errors increase linearly with the estimated duration [3]. We used a computational model of interval timing that mimics the activity of cortico-striatal structures involved in interval timing known as striatal beat frequency (SBF) model (Figure 1) [4]. In mammals, administration of DA agonist, *e.g.*, methamphetamine or cocaine produce an immediate, scalar (proportional), leftward shift in perceived time (responding earlier in time than under control conditions) whereas DA antagonist, *e.g.*, haloperidol, has an opposed effect. Upon chronic administration of DA drugs, the timing functions recalibrate, *i.e.*, they shift back to the values prior to drug administration. Once the drug is discontinued, the timing functions rebound (in a scalar manner) in the opposite direction from the initial effects of the drug, which is a signature of the clock pattern. SBF model can mimic clock patterns by adjusting the firing rates of FC neurons proportional to dopamine levels. The frequency modulation by dopaminergic drugs also produces a Gaussian-like output in SBF model. However, behavioral experiments indicate that although the average responses in PI procedure follow an almost Gaussian curve, there is always a notable skewness and a long tail of the output function. We found that frequency variability (noise) determines the deviation form the Gaussian-like output.
